# mTHPC-loaded extracellular vesicles outperform liposomal and free mTHPC formulations by an increased stability, drug delivery efficiency and cytotoxic effect in tridimensional model of tumors

**DOI:** 10.1080/10717544.2018.1513609

**Published:** 2018-12-05

**Authors:** Marie Millard, Ilya Yakavets, Max Piffoux, Amanda Brun, Florence Gazeau, Jean-Michel Guigner, Jordane Jasniewski, Henri-Pierre Lassalle, Claire Wilhelm, Lina Bezdetnaya

**Affiliations:** a CRAN, CNRS UMR 7039, Université de Lorraine, Nancy, France;; b Research Department, Institut de Cancérologie de Lorraine, Université de Lorraine, Nancy, France;; c Laboratory of Biophysics and Biotechnology, Belarusian State University, Minsk, Belarus;; d Laboratoire Matière et Systèmes Complexes, CNRS UMR 7057, Université Paris-Diderot, Paris, France;; e Laboratoire IMPMC, Université de la Sorbonne, Paris, France;; f LIBio, Université de Lorraine, Nancy, France

**Keywords:** mTHPC, photodynamic therapy, multicellular tumor spheroid, extracellular vesicles, nanocarriers

## Abstract

Efficient photodynamic therapy with *meta*-tetra(hydroxyphenyl)chlorine requires the application of specific nanoformulations. mTHPC liposomal formulation (Foslip^®^) demonstrated favorable pharmacokinetics properties. However, rapid liposomes destruction in circulation and rapid mTHPC release impedes Foslip^®^ applications. Alternatively, mTHPC nanovectorization using extracellular vesicles (EVs) could be an attractive option. EVs are naturally secreted by the organism to play a role in intercellular communication due to the capacity to transport proteins and nucleic acids. EVs also possess a natural ability to deliver therapeutic molecules into cancer cells. The aim of the present study was to evaluate photophysical and photobiological properties of mTHPC loaded in endothelial EVs as nanocarriers. We also studied efficiency of nanovectorisation on mTHPC distribution and PDT activity in multicellular tumor spheroids (MCTSs). MCTS is a nonvascularized in vitro 3D model of cells that mimics a similar microenvironment to in vivo situation. mTHPC-EVs were characterized by means of spectroscopic techniques, flow cytometry and nanoparticle tracking analysis. Compared with Foslip^®^, mTHPC-EVs are stable in murine plasma. Better mTHPC accumulation and penetration (up to 100 µm) in MCTS was observed for mTHPC-EVs compared with liposomal mTHPC. These factors could explain enhanced photodynamic activity of mTHPC-EVs compared with free and liposomal mTHPC. The light dose inducing 50% of cell death with mTHPC-EVs was 4 and 2.5-times lower than that of free and liposomal mTHPC. The obtained results demonstrate that EVs should be considered as perspective nanocarriers for mTHPC-mediated PDT.

## Introduction

Photodynamic therapy (PDT) is a good alternative to surgery for the treatment of small light-accessible tumors. Tumor eradication induced by PDT is based on the activation of photosensitizing agent, termed photosensitizer (PS), by visible light in the presence of oxygen (Dougherty, 2002; Agostinis et al., 2011). A routine use of PDT in clinic is however hampered by poor water solubility of PSs leading to aggregation, suboptimal biodistribution and unfavorable pharmacokinetics (Konan et al., 2002; Jin & Zheng, 2011).

One of the most powerful PS is temoporfin (*meta*-tetra(hydroxyphenyl)chlorin, mTHPC), marketed as Foscan^®^ and clinically approved in Europe since 2001 (Senge & Brandt, 2011). mTHPC is characterized by high absorption in the deep red (652 nm) and high singlet oxygen quantum yield (Bonnett & Martínez, 2002). Highly aggregated in biological medium and bound to plasma proteins with strong affinity, mTHPC exhibits a long half-life in the bloodstream resulting in unfavorable biodistribution and moderate skin photosensitivity (Hopkinson et al., 1999; Sasnouski et al., 2005; de Visscher et al., 2011). Such adverse characteristics resulted in the development of two liposomal formulations of mTHPC, namely mTHPC encapsulated in conventional (Foslip^®^) or PEGylated liposomes (Fospeg^®^).

As was demonstrated in preclinical models, liposomal mTHPC present several advantages such as drug monomerisation, better tumor selectivity and decreased skin accumulation (Dragicevic-Curic & Fahr 2012; Maeda et al., 2016). However, fragility in the circulation of mTHPC loaded conventional liposomes (Reshetov et al., 2012) along with a limited penetration of liposomal mTHPC in tumor tissue restrain mTHPC-based liposomal applications (Lassalle et al., 2009; Gaio et al., 2016).

Attractive candidates for efficient drug delivery could be Extracellular Vesicles (EVs) (Van der Meel et al., 2014; Vader et al., 2016). This collective term includes different subtypes usually discriminated by their size and their intracellular origin. Exosomes (30–150 nm in size), originated from multivesicular endosomes, are released upon fusion with plasmatic membrane whereas microvesicles, more heterogeneous in size (up to 1000 nm) are generated directly from plasma membrane budding. Apoptotic bodies (50–5000 nm in size) are formed by cells undergoing apoptosis. Major advantages of EVs over synthetic nanocarriers are their biocompatibility and immunotolerance. Similarly to liposomes, the EV membrane is essentially a lipid bilayer enriched by phospholipids, cholesterol and other membrane-specific lipids (van Dommelen et al., 2012). In addition, numerous proteins including those involved in membrane integrity and trafficking (Van der Meel et al., 2014), contribute to EVs natural targeting properties, their stability in blood circulation and protection of the payload from degradation (van Dommelen et al., 2012; Vader et al., 2016). It has been also demonstrated that vesicular nanocarriers significantly improved drug release and penetration into tissues in contrast to conventional rigid liposomes (Pandey et al., 2015). Different porphyrins were reported to be efficiently loaded in EVs and significantly improved cellular uptake and PDT efficiency compared with free and liposomes-encapsulated drugs with similar membrane composition (phosphatidylcholine and cholesterol) (Fuhrmann et al., 2015). EVs loaded with magnetic NPs for imaging and mTHPC for PDT were also investigated with a theranostic purpose. Compared with free mTHPC, this new class of theranosomes demonstrated improved PDT efficacy in vivo in a murine cancer model after direct intratumor injection (Silva et al., 2013b).

The understanding of the full potential of PDT with mTHPC-based EVs requires a better comprehension of multiply parameters including mTHPC intratumor diffusion, EVs-mTHPC stability in circulation and mechanism of mTHPC cellular incorporation. A comparison with liposomal-mTHPC, Foslip^®^ is also essential.

In this study, we evaluated photophysical and photobiological properties of mTHPC with EVs as nanocarriers in different experimental models and compared mTHPC-EVs with free and liposomal mTHPC. We investigated photophysical characteristics of mTHPC-EVs, their membrane integrity and stability in plasma. We further studied mTHPC-based EVs accumulation, penetration, intracellular distribution and photocytotoxicity in conventional monolayer cells and in multicellular tumor spheroids (MCTS). The use of MCTS in the studies of drug testing is rapidly increasing as MCTS better correspond to more complex and physiologically relevant cell culture model. Compared with monolayers, MCTS cultures better mimic in vivo small avascular tumors enabling the treatment optimization prior to in vivo studies (Patel et al., 2015; Millard et al., 2017; Lu & Stenzel, 2018).

## Materials and methods

### Photosensitizers

mTHPC [3,3′,3″,3″′-(2,3-dihydroporphyrin-5,10,15,20-tetrayl)tetraphenol] and its liposomal formulation (Foslip^®^) were kindly provided by biolitec research GmbH (Jena, Germany). Foslip^®^ is based on dipalmitoylphosphatidylcholine (DPPC) and dipalmitoylphosphatidylglycerol (DPPG) and mTHPC with drug:lipid ratio of 1:12 (mol/mol) and DPPC:DPPG ratio of 9:1 (w/w). For different cell experiments, stock aqueous solutions of liposomal mTHPC or ethanol solution of free mTHPC, were diluted in cell culture medium supplemented with 2% heat-inactivated fetal calf serum (FCS) to obtain final concentrations of either 0.1 µM or 1.45 µM for monolayer cells and 3.6 µM for spheroids cells. Final ethanol concentration in medium never exceeded 0.1%.

### Production of mTHPC-EVs

Human umbilical vascular endothelial cells (HUVEC) were cultured as described earlier in DMEM endothelial medium (Gibco^®^, United Kingdom) containing 9% FCS at 37 °C (5% CO_2_, humidified atmosphere) (Silva et al., 2013a). At near-confluence of cells, the medium was replaced by a 5 µM mTHPC in serum-free phenol red-free Roswall Park Memorial Institute (RPMI)-1640 medium (Invitrogen, Cergy-Pontoise, France) for 2 h at 37 °C. After washing with free RPMI, cells were maintained in this medium during 3 days at 37 °C to induce EVs release according to the method used previously (Silva et al., 2013b) with slight modifications. Briefly, the culture supernatant was centrifuged (2000*g* for 10 min) to eliminate cell debris and apoptotic bodies. After ultracentrifugation (100,000*g* for 1 h 10 min at 4 °C), EVs were characterized by nanoparticle (NP) tracking analysis (NTA 3.2 Software, Malvern Instruments, UK). mTHPC concentration was estimated with LS55 spectrofluorometer (Perkin Elmer, USA).

Flow cytometry analysis using antibodies (BD Biosciences, Le Pont de Claix, France) was conducted to characterize EVs. Exosomes were highlighted using established exosomal markers (CD9, CD63 and CD81) stained with phycoerythrin (PE); microvesicles were distinguished using endothelial membrane markers stained with fluorophores (CD31-FITC, CD144-PE). Phosphatidylserines, present on the microvesicle surface, were revealed by Annexin V-FITC staining.

### Spectroscopic measurements

Absorption measurements were recorded with a Lambda 35 spectrometer (Perkin Elmer, USA) using integrating sphere and fluorescence measurements were conducted with LS55B spectrofluorometer (PerkinElmer, USA) equipped with polarizers, thermostated cuvette compartments and magnetic stirring for polarization experiments. Fluorescence quantum yield and photoinduced fluorescence quenching (PIQ) were measured as was previously described (λexc: 416 nm; λem: 652 nm) (Reshetov et al., 2011). mTHPC fluorescence polarization was performed as described earlier (Reshetov et al., 2011). Samples were excited at 435 nm and fluorescence was registered at 652 nm.

### Size distribution, stability and transmission electron microscopy (TEM)

Size distribution profiles and stability of mTHPC-EVs were obtained using Nanosight LM10-T14 system (Malvern Instruments, UK) equipped with a 532 nm laser at 50 mW and the sCMOS camera. For NTA, mTHPC-EVs were diluted in PBS (1:5000). Each of six samples from different isolations was recorded five times for 30 s with temperature regulated at 25 °C. Particle diameter was calculated from Stokes-Einstein equation with NTA. For stability experiments, EVs-mTHPC and Foslip^®^ were incubated with 20% of exo-free murine plasma in PBS at 37 °C up to 24 h. At different times, 100 µL of sample was diluted in PBS and cooled down to 4 °C in the dark until NTA measurements.

Gel migration was performed with mTHPC-EVs or Foslip^®^ (at 10 µM of mTHPC) incubated in 20% exo-free murine plasma. Twenty microliter of samples were deposited in each well with 2 µL of blue juice on agarose gel (1% agarose and 10% Tris, Borate, EDTA buffer 10×). Migration was performed during 3 h at 100V. Control was realized with a solution of free-mTHPC in 0.3% triton-PBS.

For Cryo-TEM experiments, mTHPC-EVs and Foslip^®^ were placed in PBS containing 20% of exo-free murine plasma at 37 °C for 6 and 24 h. Controls were realized without plasma. Five microliter droplets of samples were deposited on Quantifoil^®^ (Quantifoil MicroTools GmbH, Germany) carbon membrane. After removing the excess of liquid with a filter paper, the membrane was rapidly immersed in liquid ethane cooled in liquid nitrogen. The samples were transferred to the microscope and observed at –180 °C using a LaB_6_ JEOL JEM2100 (JEOL, Japan) cryomicroscope operating at 200 kV with a JEOL low dose system (Minimum Dose System, MDS).

### Monolayer cell culture

HT29 human colon adenocarcinoma cells (ATCC^®^, LGC Promochem, Molsheim, France) were grown at 37 °C (5% CO_2_, humidified atmosphere) in RPMI medium and in minimum essential media (Gibco^®^, United Kingdom), respectively. Both medium were supplemented with 9% FCS and 1% 200 mM l-glutamine (Life Technologies, Carlsbad, USA).

### MCTS formation

MCTS were initiated as previously described (Marchal et al., 2005). Briefly, flasks coated with 1% l-agarose were seeded with 5 × 10^4^ HT29 cells/mL. After three days, cellular aggregates were transferred into spinner flasks (Integra Biosciences) containing 125 mL RPMI medium. Spinner flasks were placed under constant agitation at 75 rpm at 37 °C (5% CO_2_, humidified atmosphere) during 15 days. Spheroids were filtered to obtain ∼500 µm in diameter before conducting experiments.

### Cellular uptake

#### Monolayer cells

HT29 cells (5 × 10^4^ cells/mL) were seeded in 24-well plates for 72 h and incubated with free mTHPC, Foslip^®^ or mTHPC-EVs (0.1 µM) during 3, 6 or 24 h. After three washings with PBS, chemical extraction was performed. Cells were trypsinized and absolute ethanol was added to cell pellet. After sonication (15 min) and centrifugation (5 min, 1500 rpm), the fluorescence of supernatant containing mTHPC was assessed (λexc: 416 nm; λem: 652 nm). mTHPC concentrations were determined by using a calibration curve obtained from a standard mTHPC solution in absolute ethanol. Results were normalized to the quantity of proteins measured by DC protein assay (Biorad).

#### Spheroid cells

Fifty spheroids were collected and washed three times before incubation with mTHPC formulations (3.6 µM) for 3, 6 or 24 h. After three washings with PBS, chemical extraction of mTHPC was performed as previously described for monolayer cells. Results were normalized to the number of spheroids.

### Mechanism of nanocarriers uptake

HT29 cells (5 × 10^4^ cells/mL) were seeded in 24-well plates for 72 h, and mTHPC formulations (1.45 µM) were added to cells at 4 °C for 1 h. After three washings with PBS, cells were pre-incubated for 30 min at 37 °C with medium containing either 100 µM EIPA or 400 µM genistein or 10 µg.mL^−1^ chlorpromazine. Free mTHPC, Foslip^®^ and mTHPC-EVs (1.45 µM) were added for 1 h in the dark at 37 °C. Cell extraction was realized as described above. Results were normalized to mTHPC uptake obtained for control group (37 °C, without inhibitors).

### Confocal microscopy

#### Subcellular colocalization

HT29 cells (1.5 × 10^4^ cells/mL) were seeded into Slideflasks (Nunc, Roskilde, Denmark). After 72 h, mTHPC formulations (1.45 µM) were incubated with cells for 24 h and after three washings in PBS, organelle specific fluorescent probes were added: mitochondria and lysosomes were labelled at 37 °C for 30 min with 0.2 µM of MitoTracker^®^ Green or 0.15 µM of LysoTracker^®^, respectively (Molecular Probes, OR); endoplasmic reticulum (ER) was labelled at room temperature for 1 min with 2.5 µg/mL of DiOC_6_ whereas Golgi apparatus was labelled with 5 µM of NBD-C_6_ ceramide for 30 min at 4 °C followed by 30 min at 37 °C. After three washings, fluorescence was observed with a confocal laser-scanning microscope (Leica SP5 X AOBS LCSM, Leica microsystem, Wetzlar, Germany). Organelle-specific fluorescent probes were excited at 488 nm and mTHPC at 520 nm. Band-pass emission filters of 505–550 and 635–700 nm were, respectively, used to discriminate organelles probes (channel 1, green) from mTHPC (channel 2, red) fluorescence. Fifteen different focal planes observed with a water immersion objective (X63) were acquired (512*512 pixels) and the LAS Life software (Leica microsystem, Wetzlar, Germany) was used to obtain maximum projection images. Colocalisation was quantified with Pearson’s correlation coefficient (PCC) by ImageJ software.

#### mTHPC diffusion inside spheroids

Thirty spheroids were collected and washed three times before 24 h incubation with mTHPC formulations (3.6 µM). After three washings, spheroids were frozen in Tissue-Tek^®^ O.C.T.™ and 14 µm thick sections were used for confocal microscopy. Band pass emission filters were set as described above. Imaging of mTHPC diffusion was performed at the central section of the spheroid (the one corresponding to the largest diameter) and image acquisition of this section was done on 20 different focal planes in 1024*1024 pixels by the LAS AF Life software to obtain maximum projection images. Ten radial lines (regions of interest, ROI) were randomly drawn in the maximum projection images to obtain fluorescence profiles. The fluorescence profile of each spheroid as a function of depth was determined with the ImageJ software. The percentage of fluorescence intensity (relative to peak intensity) found at different radial distances from the outer spheroid periphery was used to obtain mTHPC penetrative capacity.

### Photodynamic treatment

#### Monolayer cells

HT29 cells (4 × 10^4^ cells/mL) were seeded in 96-well plates for 72 h. mTHPC formulations (0.1 µM) were incubated with cells for 24 h. After washing, cells were irradiated at 652 nm with a diode laser (Cerelas, Biolitec GmbH, Germany) at different fluences (fluence rate 4.5 mW/cm^2^), medium was replaced by the fresh one supplemented with 9% FCS, 24 h later, cellular viability was evaluated by MTT test as described previously (Marchal et al., 2005). Photocytotoxicity was evaluated using LD_50_, defined as the light dose inducing 50% of cell death.

#### Spheroid cells

Fifty spheroids were collected and washed three times before incubation with mTHPC formulations (3.6 µM) for 24 h. After that, spheroids were transferred into 35 mm Petri dishes containing 3 mL of medium and irradiated at 652 nm with different fluences at the fluence rate of 30 mW/cm^2^. Control spheroids were exposed to culture medium only (no drug, no light) or to free mTHPC only (drug, no light). Immediately after irradiation, cell survival assay was performed as previously reported (Marchal et al., 2005). Briefly, spheroids were dissociated and 500 cells were seeded in triplicate into 6-well plates. After 10 days, cell colonies fixed in 70% ethanol were stained with 1% crystal violet for 5 min. Cell survival was normalized to the number of colonies (more than 50 cells) obtained from control spheroids.

### Immunohistochemistry (IHC) for apoptosis evaluation

Thirty spheroids were irradiated (30 mW/cm^2^, 30 J/cm^2^) as indicated above and 6 h after treatment, spheroids were fixed with 4% formaldehyde for 16 h and embedded in paraffin to realize 4-µm-thick sections. Apoptotic cells staining by IHC was performed as described earlier (Bressenot et al., 2009) with slight modifications. Briefly, sections were incubated in 10 mM Tris/EDTA solution (pH = 8) at 98 °C for 10 min. Endogenous peroxidase activity was blocked in a 3% hydrogen peroxide solution for 5 min and after washings in PBST, primary antibodies (diluted 1:100) were added for 1 h at room temperature. The biotinylated secondary antibody (diluted 1:200) was applied after washing for another 1 h. Sections were then washed and incubated in streptavidin-peroxydase for 30 min at room temperature. After washings, bound peroxidase was identified using the NovaRED system and nuclear counterstaining with hematoxylin coloration was performed. The apoptotic cell index was calculated as the number of labelled cells per total number of cells in spheroid section.

### Statistics

The data from at least three independent experiments are presented as mean ± standard deviation. The data were evaluated using nonparametric Mann Whitney’s *U* test (StatView™ software) with a significant level of *p* < .05.

## Results

### Characterization of mTHPC-EVs

**Figure 1.**
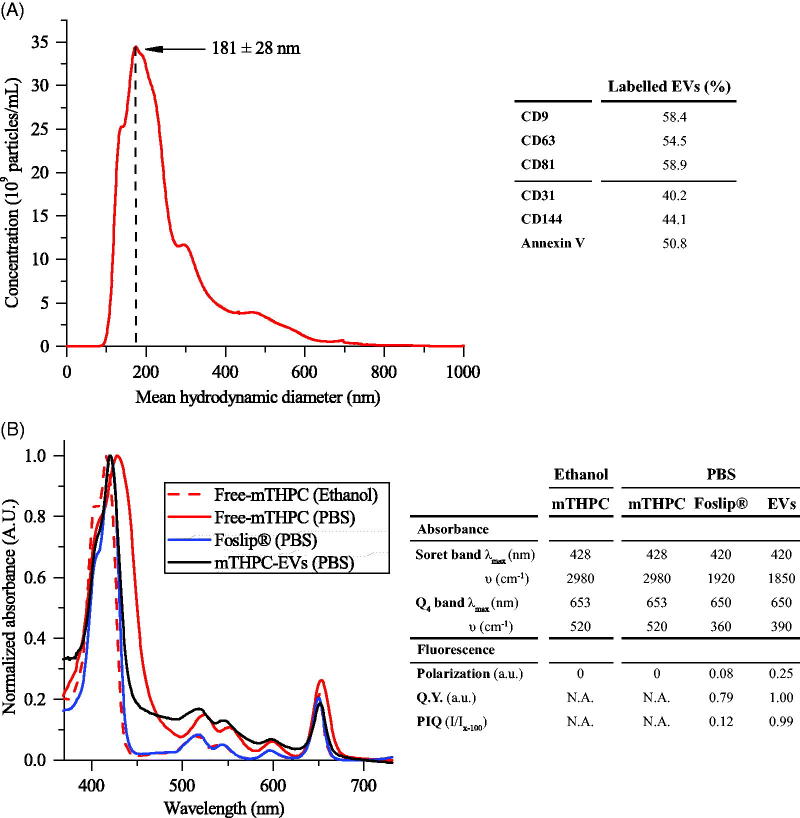
mTHPC-loaded nanocarriers characterization. (A) Size distribution profile of mTHPC-EVs, obtained by NTA (left panel) and repartition of exosomes and microvesicles in mTHPC-EVs population by flow cytometry analysis (right panel) using specific antibodies. (B) Normalized absorption spectra (left panel) of free mTHPC in ethanol and PBS, Foslip® in PBS and mTHPC-EVs in PBS. mTHPC concentration was 1.45 µM. Right panel displays photophysical parameters derived from absorption spectra. λmax (nm): maximal wavelength of absorption; υ (cm^–1^): half height bandwidth; F.Y: fluorescence yield; *I*/*I*_x-100_: normalized fluorescence (exposure 500 mJ/cm^2^).

mTHPC-EVs were characterized by NTA and the mean hydrodynamic diameter of EVs was 181 ± 28 nm (Figure 1(A)). The surface charge was negative with a Zeta potential value of (–15) mV. After isolation procedure, the concentration of mTHPC in EV stock solution, determined spectroscopically, was 98 ± 22 µM. NTA assessed EVs density was further estimated as 4 × 10^12^/mL. The composition of mTHPC-EVs was assessed by flow cytometry using conventional exosomal markers (CD9, CD63 and CD81) and microvesicles markers, including CD31 and CD144, which correspond to EVs derived from endothelial cells membrane and phosphatidylserine (Annexin-V). As follows from Figure 1(A), mTHPC-EVs have revealed a roughly similar proportion of exosomes and microvesicles with a slight prevalence of exosomes.

### Photophysical properties of mTHPC-EVs

Spectroscopic characteristics of mTHPC-based different formulations are displayed in Figure 1(B). In ethanol, the absorption spectrum of monomerized mTHPC is characterized by Soret band (maximum at 416 nm) and four Q-bands with a prominent peak at 650 nm (Figure 1(B), right panel). Absorption spectra of Foslip^®^ and mTHPC-EVs in PBS solution were very close to that of mTHPC in ethanol, thus indicating that mTHPC is present in monomeric form in both formulations (Figure 1(B)).

Relative fluorescence quantum yield of mTHPC embedded in EVs was comparable with mTHPC ethanol solution and was 20% higher than that of Foslip^®^ (Figure 1(B)). There are several parameters characterizing mTHPC microenvironment in nanoformulations, such as fluorescence polarization (p) and PIQ of fluorescence (*I*/*I*_x-100_). Consistent with our previous study (Reshetov et al., 2011), the (p) degree of Foslip^®^ was 0.082 and PIQ degree was 0.12 (Figure 1(B), right panel). At the same time, mTHPC-EVs display increased polarization compared with Foslip^®^ (0.251 vs 0.082) and absence of PIQ (Figure 1(B), right panel). As we demonstrated earlier (Reshetov et al., 2011), a decrease of mTHPC content to 1:600 dye:lipid ratio leads to the disappearance of the photoinduced response (PIQ approaching 1). Based on these photophysical properties, we can indirectly estimate the loading capacity of mTHPC in EV (ratio mTHPC:lipid) as 1:600, while the direct loading of mTHPC in lipid bilayer of Foslip^®^ was as high as 1:12.

### Nanocarrier stability in murine plasma

**Figure 2.**
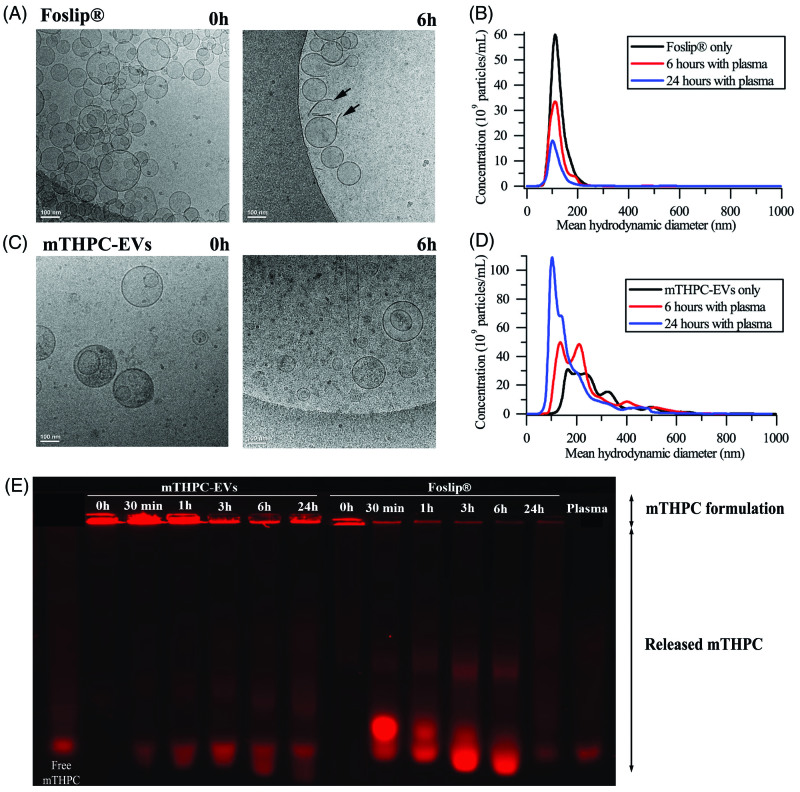
Cryo-TEM images of (A) Foslip^®^ and (C) mTHPC-EVs. Arrows show membrane fragments of Foslip^®^. Scale bar: 100 nm. mTHPC concentration was 10 mM. Histograms of (B) Foslip^®^ and (D) mTHPC-EVs particles distribution obtain by NTA. (E) Fluorescence profile of mTHPC formulations after migration in agarose gel showing mTHPC leakage from nanocarriers. Nanocarriers were incubated in PBS (0 h) or 20% murine plasma at 37 °C. mTHPC concentration was 20 µM.

Three different techniques were employed to assess structural stability of different formulations in plasma: cryo-TEM, NTA and gel migration. Incubation of mTHPC-EVs and Foslip^®^ in PBS without serum (24 h, 37 °C) did not reveal variations of particle concentration (data not shown). Already after 6 h incubation with plasma proteins, Foslip^®^ were destructed, as evidenced by the presence of numerous membrane fragments (arrows in Figure 2(A)). The quantitative analysis, obtained by NTA, showed that the size of intact liposomes remained unchanged (120 nm) until 24 h (Figure 2(B), Supplementary Material Figure S1A). Unexpected behavior was observed for mTHPC-EVs. During the whole observation period, mTHPC-EVs conserved membrane integrity along with a significant reduction of EVs size (Figure 2(C), Supplementary Material Figure S1B). This result was further refined by NTA and showed that the mean hydrodynamic diameter of mTHPC-EVs decreased from 160 nm at the beginning of experiment to 60 nm after 24 h. At the same time, we registered a significant increase in the number of EVs (1.6-times after 6 h and 3.6-times after 24 h) (Figure 2(D), Supplementary Material Figure S1B).

We further assessed mTHPC release from nanocarriers using gel migration. mTHPC-EVs in murine plasma showed much less release of mTHPC to serum proteins compared with Foslip^®^ (Figure 2(E)). Considering the fluorescence intensity of each well, we can deduce that more than 50% of mTHPC was release from liposomes at already 30 min after incubation whereas it takes about 6 h for EVs, thus pointing out to mTHPC-EVs stability in plasma enriched solution.

### mTHPC uptake in monolayer cells

#### mTHPC accumulation

Until 6 h incubation, cellular uptake of mTHPC-EVs was similar to that obtained with other mTHPC formulations. From 6 to 24 h, mTHPC-EVs uptake was increased by a factor of 12 and after 24 h mTHPC-EVs intracellular concentration was 1.7-times higher than that with free mTHPC and Foslip^®^ (0.51 ng/mg protein and 0.48 ng/mg protein, respectively) (Supplementary Material Figure S2).

#### Subcellular colocalization

**Figure 3.**
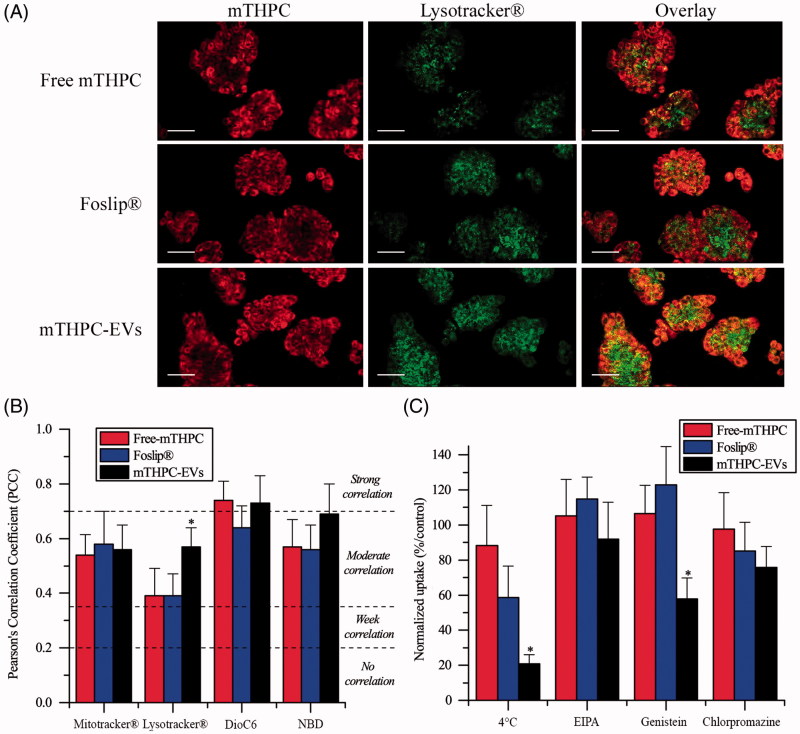
(A) Typical confocal images HT29 cells costaining with different mTHPC formulations (1.45 µM) and Lysotracker^®^. Left panel displays mTHPC images, center panel displays Lysotracker^®^ images and right panel displays overlay images. These images were representative of three independent experiments. Scale bar: 50 µm (B) PCC values obtained from overlay images. (C) Endocytosis pathways inhibition of cellular uptake of mTHPC formulations after incubation at 4 °C or 37 °C with endocytosis inhibitors. *: *p* < .05 compared to free and liposomal mTHPC.

Subcellular localization of mTHPC was evaluated by confocal microscopy after 24 h incubation with cells (Figure 3(A), Supplementary Material Figure S3). All mTHPC formulations displayed almost homogeneous distribution in cytoplasm outside both nucleus and plasma membrane (Figure 3(A), Supplementary Material Figure S3 left panel). Specific organelles probes were applied to evaluate subcellular localization (Figure 3(A), Supplementary Material Figure S3 center panel). Co-stained microscopy images (Figure 3(A), Supplementary Material Figure S3 right panel) were further used to compute PCC (Figure 3(B)). All mTHPC formulations showed a strong correlation with ER probe (PCC >0.7) and a moderate correlation (PCC >0.5) with mitochondrial and Golgi apparatus probes (Supplementary Material Figure S3, Figure 3(B)). The major difference was demonstrated for lysosomal localization (Figure 3(A)). PCC of mTHPC-EVs increased significantly compared with free mTHPC and Foslip^®^ (0.57 vs 0.39, *p* < .05) suggesting endocytosis pathway for mTHPC-EV intracellular incorporation (Figure 3(B)).

#### Inhibition of endocytosis pathways

Considering that mTHPC-EVs demonstrated a significantly better localization of mTHPC in lysosomes compared with other formulations, we further assessed inhibition of different endocytosis pathways (Figure 3(C)). Endocytosis of mTHPC-EVs was firstly evidenced by significant decrease in mTHPC uptake (that dropped to 34%) when cells were exposed to mTHPC-EVs at 4 °C for 1 h. We examined the mechanisms of mTHPC-EVs endocytosis using specific inhibitors as EIPA, genistein and chlorpromazine that target, respectively, micropinocytosis, caveolae and clathrin-dependent endocytosis. Cellular uptake of mTHPC-EVs was not affected by EIPA treatment but was significantly reduced (by 50%) after preincubation with genistein (*p* < .05) and to a lesser extent (by 25%) after preincubation with chlorpromazine. These results suggest the predominance of caveolae-dependent mechanism of endocytosis together with a strong probability of clathrin involvement. At the same time, cellular uptake of free mTHPC was unaffected neither at 4 °C or in the presence of specific inhibitors. No specific inhibition of endocytosis was observed for Foslip^®^, while at 4 °C, we noted a decrease of uptake by 40%, albeit it was not significant compared with free mTHPC (Figure 3(C)).

### mTHPC uptake in spheroid cells

#### mTHPC accumulation

**Figure 4.**
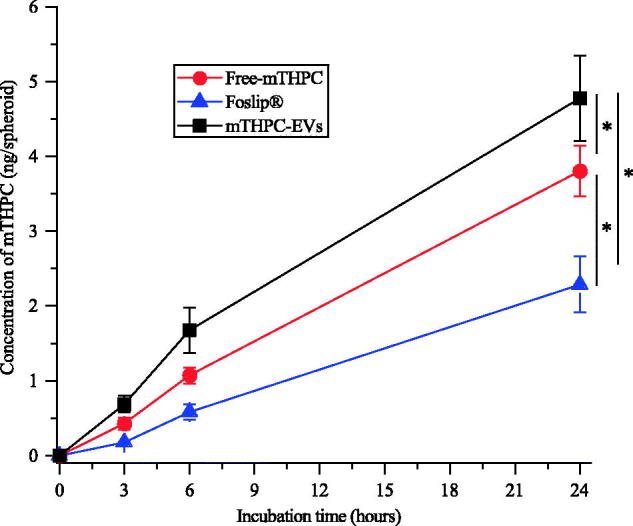
Kinetics of mTHPC uptake in HT29 spheroids after incubation with free mTHPC, Foslip^®^ and mTHPC-EVs. mTHPC concentration was 3.6 µM. Chemical extraction of mTHPC was realized in absolute ethanol. *: *p* < .05.

mTHPC concentration in spheroids was measured after 3, 6 and 24 h incubation with mTHPC-EVs, free mTHPC and Foslip^®^. Irrespective of the compound, kinetics revealed similar profiles of mTHPC accumulation with progressive increase until 24 h (Figure 4). Already after 3 h incubation, significantly higher mTHPC accumulation was observed with mTHPC-EVs (0.7 ng/spheroid) compared with 0.4 ng/spheroid for free mTHPC and 0.2 ng/spheroid for Foslip^®^ (*p* < .05). At the end of incubation period, mTHPC-EVs provided mTHPC intraspheroid concentration of 4.8 ng/spheroid, which was twice higher compared with Foslip^®^ and 1.3-times higher than that for free mTHPC (2.3 and 3.7 ng mTHPC/spheroid, respectively).

#### mTHPC diffusion inside spheroids

**Figure 5.**
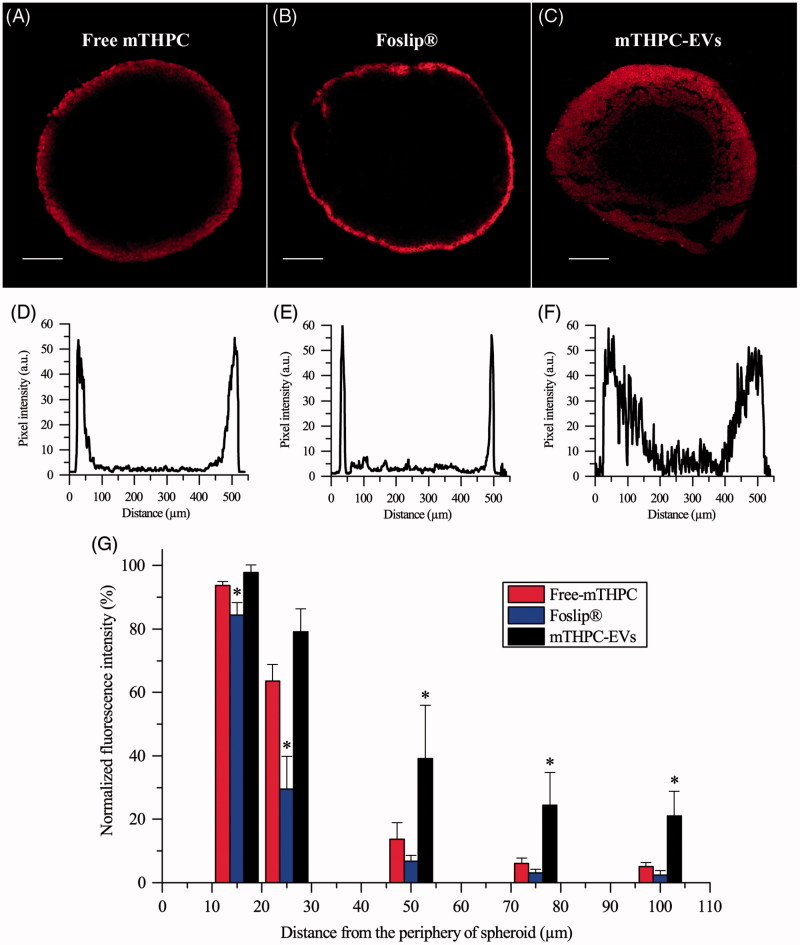
Typical fluorescence images and corresponding fluorescence profiles of spheroids after 24 h incubation with (A, D) free mTHPC, (B, E) Foslip^®^ and (C, F) mTHPC-EVs. mTHPC concentration was 3.6 µM. Scale bar: 100 µm. (G) Based on fluorescence plot profiles, normalized fluorescence was computed in a function of the distance from the periphery of spheroids. *: *p* < .05 compared to free mTHPC.

Confocal microscopy was used to evaluate mTHPC diffusion inside spheroids (Figure 5). Images revealed identical maximum fluorescence intensity at the spheroid periphery for all formulations with apparent differences in fluorescence repartition from the periphery to the spheroid core (Figure 5(A–C)). The corresponding profiles (Figure 5(D–F)) indicated that the fluorescence of free and liposomal mTHPC was limited to the external rim of the spheroid while fluorescence in the internal cell layers of the spheroid was detectable only for mTHPC-EVs. These observations were supported by the decrease of fluorescence intensity observed from the periphery to the spheroid core (Figure 5(G)). A sharp drop of fluorescence intensity was observed for both free and liposomal mTHPC at already 50 µm from periphery with a barely detectable signal at 100 µm. As for mTHPC-EVs, a gradual decrease of fluorescence was registered until 100 µm from the periphery resulting in a 22% of remaining mTHPC fluorescence detectable at this depth (Figure 5(G)).

### Photocytotoxicity of mTHPC

#### Cell survival after PDT in monolayer cells and spheroids

No significant dark cytotoxicity was observed after 24 h incubation of monolayer cells or spheroids with all mTHPC formulations. Monolayer cells incubated with mTHPC remained intact after 24 h incubation. The dark cytotoxicity of spheroids treated with EVs was 88.3 ± 3.1% and was similar to that of free mTHPC and Foslip^®^ (96.7 ± 4.5% and 97.0 ± 2.5%, respectively). Red light irradiation induced a significant decrease in cell survival in function of applied fluence (Supplementary Material Figure S4). Two major observations emerge from dose-response experiments in both cell models.

**Figure 6.**
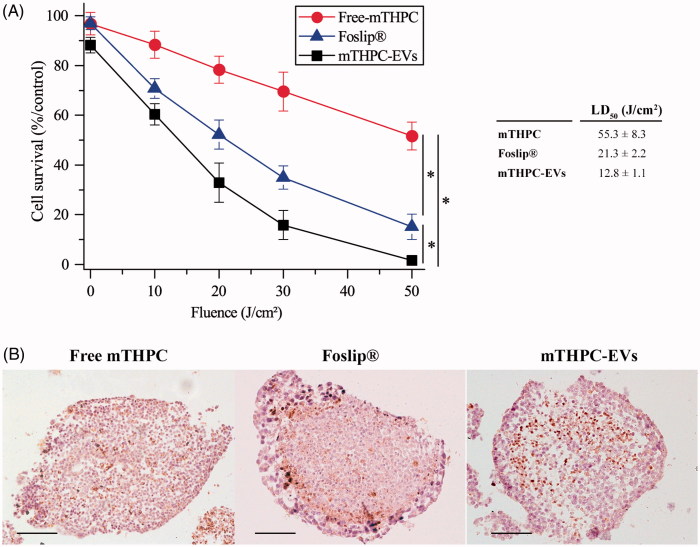
Photocytotoxicity of mTHPC formulations (3.6 µM) after 24 h incubation with spheroids. (A) Cell survival in function of applied fluence (fluence rate 30 mW/cm^2^). *: *p* < .05. On the insert, the light dose inducing 50% of cell death (LD_50_) is presented for each formulation. (B) IHC images of active caspase-3 staining (×10) in spheroids loaded with different mTHPC formulations and assessed 6 h post irradiation (30 mW/cm^2^; 30 J/cm^2^). These images are representative of three independent experiments. Scale bar: 100 µm.

Firstly, free mTHPC was by far less photocytotoxic than mTHPC loaded in EVs or liposomes in monolayer (Supplementary Material Figure S4) and in spheroid cells (Figure 6(A)). In monolayer cells, the LD_50_ was the same for Foslip^®^ and mTHPC-EVs (3.1 J/cm^2^) and nearly 2.5-times lower than that for free mTHPC (1.3 J/cm^2^) (insert to Supplementary Material Figure S4). In spheroid cells, the LD_50_ for free mTHPC was significantly higher than for Foslip^®^ (55.3 J/cm^2^ vs 21.3 J/cm^2^). The highest photocytotoxicity was noted for mTHPC-EVs with LD_50_ as 12.8 J/cm^2^) (insert to Figure 6(A)).

Secondly, mTHPC-EVs and Foslip^®^ displayed comparable pattern of cell death in 2D cells while photocytotoxicity of mTHPC-EVs was nearly twice higher in spheroids compared to that of Foslip^®^ (Figure 6(A)).

#### Photoinduced apoptosis in spheroids

Figure 6(B) shows the distribution of apoptotic cells detected by active caspase-3 staining after photodynamic treatment at 30 J/cm^2^. A loss of cell cohesion was observed after irradiation of all mTHPC formulations. The distribution patterns of apoptotic cells after PDT with free mTHPC or mTHPC-EVs (Figure 6(B), left and right panel) were similar and showed a diffusive distribution of apoptotic cells between the periphery and the necrotic core. This distribution was also demonstrated for drug only treated spheroids (Supplementary Material Figure S5) and was consistent with our previous data on residual apoptotic cells in spheroids treated with mTHPC only (Bressenot et al., 2009). In contrast, spheroids exposed to Foslip^®^/PDT displayed a large number of apoptotic cells located preferentially at the periphery of spheroid (Figure 6(B), center panel).

In control (nonirradiated spheroids), the apoptotic index (AI) was about 15% irrespective of mTHPC formulation and this percentage was not significantly increased upon PDT with free mTHPC (AI = 17.4 ± 2.8%, Supplementary Material Table S1). In contrast, we observed 1.5-times more apoptotic cells for Foslip^®^/PDT (AI = 26.3 ± 4.6%) and 2-times for mTHPC-EVs/PDT (AI = 32.4 ± 7.3%) (Supplementary Material Table S1).

## Discussion

EVs possess negligible immunogenicity, excellent bioavailability and are natural drug delivery vehicles contrary to synthetic nanovectors as liposomes (van Dommelen et al., 2012). In addition, membrane composition of EVs allows increasing drug delivery efficiency to targeted cells (Vader et al., 2016; Kim & Kim, 2017). As was shown recently, the EVs produced from HUVEC demonstrated both increased stability and cell-binding ability. These properties were attributed to the HUVEC membrane composition rich in cholesterol, sphingomyelin (SM) and proteins compared with cholesterol-free liposomes (Fuhrmann et al., 2015). The loading of these HUVEC-derived EVs with different porphyrins resulted in PSs better intracellular uptake compared with liposomal PS formulations. Therefore, favorable properties of mTHPC loaded in HUVEC derived EVs were anticipated. Indeed, much better stability (Supplementary Material Figure S1) and intracellular accumulation (Supplementary Material Figure S2) was demonstrated for mTHPC-EVs compared to mTHPC liposomal formulation.

Spectral properties of mTHPC in different formulations are summarized in Figure 1(B) (right panel). mTHPC is prone to aggregation in aqueous solvent forming large aggregates. Similar to other studies (Belitchenko et al., 1998; Kascáková et al., 2008), mTHPC aggregation was characterized by decreased extinction coefficients and bathochromic shifts of the main peaks (416–428 nm and 650–653 nm) (Figure 1B). mTHPC embedding into liposomal nanovectors prevents PS aggregation (Reshetov et al., 2012). Absorption spectra of both liposomal and EVs-mTHPC were very close to monomeric mTHPC solution in ethanol (Figure 1(B), left panel) thus clearly indicating monomeric state of mTHPC in EVs.

The loading capacity of NPs could be deduced from PIQ experiments (Reshetov et al., 2011). mTHPC-EVs provided fluorescence quenching 0.99 (Figure 1(B), right panel), which is close to that (0.96) previously shown for mTHPC loaded into liposomes at the ratio 1:600, being significantly lower than that for Foslip (1:12) (Reshetov et al., 2011). This low loading capacity of EVs could be related to the production method of EVs. As was recently demonstrated, the loading of cells with the drug before EVs production is less effective compared with drug loading after the EVs production (Vader et al. 2016). Indeed, as was shown in the article of Fuhrmann et al. (2015), the loading efficiency was 8 times higher when EVs were loaded with porphyrins after EV production compared to passive loading (Fuhrmann et al., 2015).

While in circulation, phosphatidylcholine vesicles are accommodated by lipoproteins ceasing to exist as identifiable entities (Damen et al., 1981; Bonté & Juliano, 1986). Earlier, we observed this rapid destruction of DPPC/DPPG liposomes, empty or loaded with mTHPC (Foslip^®^) in the presence of plasma proteins (Reshetov et al., 2012). As can be seen in the Figure 2(A), there are the pin-shaped fragments of liposomes after 6 h of incubation of Foslip^®^ with plasma. EVs exhibit the behavior completely different to Foslip^®^. According to the data obtained with cryo-TEM and NTA, incubation of EVs with murine plasma leads to the decrease of large size vesicles (>150 nm) with the subsequent increase of small size vesicles (60 nm) (Figure 2(D); Supplementary Material Figure S1B). Several factors could affect EVs stability against plasma proteins.

Firstly, EVs contains membrane proteins (tetraspanins, cell-specific receptors, …), which interact with near located lipids forming the lipid rafts (Rosa-Fernandes et al., 2017). Lipid rafts restrict interaction of plasma components with EVs thus protecting vesicles from degradation (Maas et al., 2017).

Further, EVs membrane produced from HUVEC cells is rich in cholesterol (23.5%) and SM (10.3%) (Fuhrmann et al., 2015), both of which significantly increase stability of vesicles in serum due to the inhibition of lipid transfer to High Density Lipoprotein/Low Density Lipoprotein (Hernández-Caselles et al., 1993). Following removal of lipids from non-raft domains to lipoproteins, the raft lipids like cholesterol and SM as well as membrane proteins get concentrated. At the end, the surface of small EVs is made almost exclusively of membrane proteins that can protect the surface by steric repulsion. As a result, we observed a decrease of EVs size (Figure 2, Supplementary Material Figure S1). Moreover, the fractionations of EVs upon the interaction with serum proteins could not be excluded.

In monolayer cells, mTHPC-EVs showed two times higher photocytotoxicity than free mTHPC. Similar results were obtain with exosomes charged with acridine orange (AO) due to a better cellular uptake of AO-loaded exosomes compared with free AO (Iessi et al., 2017). Unexpectedly, no difference in photocytotoxicity was observed between liposomal mTHPC and mTHPC-EVs despite better mTHPC uptake after cells incubation with mTHPC-EVs (Supplementary Material Figure S5). A possible explanation could be an important lysosomal localization of mTHPC-EVs compared to free mTHPC and Foslip^®^ (PCC: 0.57 vs 0.39) (Figure 3(B)). Lysosomes are organelles involved in degradation of materials, resulting in a partial drug inactivation. In consequence, a subcellular localization of mTHPC in lysosomes is less favorable in terms of photocytotoxicity (Teiten et al., 2003).

Considering that mTHPC-EVs displayed an important localization in lysosomes, a study of endocytosis pathways was realized and showed that mTHPC-EVs were incorporated in cells preferentially by endocytosis (Figure 3(C)). This subcellular localization is in favor of clathrin dependent endocytosis (Sahay et al., 2010). However, our results obtained by using specific inhibitors indicated that only 25% of internalization in cells is due to a chlatrin dependent mechanism. The major uptake mechanism of EVs could be attributed to the caveolae dependent pathway (Figure 3(C)). This phenomenon was already reported for polymeric micelles that bypass endosomes and are further transported to lysosomes. Authors suggested that endocytosis pathways can be deregulated in cancer cells resulting in a nanocarrier transportation to lysosomes by caveolae (Sahay et al., 2010). Similar to other observations (Peng et al., 2014), free mTHPC uptake was not much affected by 4 °C thus indicating a passive diffusion mechanism. At the same time at 4 °C, uptake of Foslip^®^ was inhibited by 40% while uptake in the presence of specific inhibitors was unaffected (Figure 3(C)). This could indicate incorporation of Foslip^®^ by fusion of liposomes with plasma membrane. Batzri and Korn have demonstrated that uptake by fusion was inhibited almost completely at low temperature (Batzri & Korn, 1975). Two other mechanisms could be involved in Foslip^®^ uptake, namely a diffusion of mTHPC from the liposomes to the plasma membrane (Hefesha et al., 2011) and transport of mTHPC by lipoproteins after liposome destruction and PS redistribution (Kiesslich et al., 2007).

MCTS are known to be a suitable model to study PDT parameters and to evaluate the penetration of nanodrugs. mTHPC shows a high sequestration in cells and therefore, displays an unhomogeneous distribution inside MCTS (Foster et al., 1993; Gaio et al., 2016). Consistent with this observation, free mTHPC was confined at the spheroid periphery and fluorescence intensity was decreased 8–10 times at already 50 µm in depth (Figure 5(A,D)). The same fluorescence pattern has been demonstrated for Foslip^®^ (Figure 5(B,E)). This result is consistent with numerous studies, where mTHPC embedded in liposomes, PLGA or solid lipid NPs did not enhance mTHPC diffusion in MCTS compared with free mTHPC (Löw et al., 2011; Gaio et al., 2016; Hinger et al., 2016). Authors suggested that the physicochemical properties of NPs are important for its transport and distribution in MCTS. Contrary to liposomal mTHPC, mTHPC-EVs display an improvement of mTHPC diffusion inside spheroid (Figure 5(F,G)). This phenomenon can be explained by the decrease of mTHPC-EV size during incubation (160–60 nm at 24 h incubation) (Figure 2(D), Supplementary Material Figure S2). It was demonstrated that small NPs (*ca.* 50 nm in diameter) showed a better penetration into spheroid compared to NPs with a diameter at 120 nm (Hinger et al., 2016; Millard et al., 2017). Another explanation could be related to the capacity of EVs to fuse with cell membrane and penetrate inside the spheroid by successive rounds of EVs uptake (Lee et al., 2015). The authors suggested that EVs were incorporated into the first cell layer of spheroid and transferred their cargo into neighboring cells by production of new EVs (Lee et al., 2015).

A better intracellular uptake was also demonstrated for mTHPC-EVs compared with other formulations (Figure 4). As was shown earlier exosomes loaded with AO displayed an improved incorporation and longer retention in melanoma spheroids compared with free AO. This observation was attributed to the membrane composition of exosomes creating electrostatic and lipid-lipid interactions with cells resulting in improved AO retention (Iessi et al., 2017).

Impact of mTHPC encapsulation on PDT efficiency was evaluated by clonogenic assays (Figure 6(A)). As anticipated a better photocytotoxicity was demonstrated with mTHPC-EVs loaded spheroids compared with other formulations (Figure 6(A)). A complex relationship beteween cytotoxicity, drugs uptake and drugs diffusion into spheroids was already highlighted in the article of Solomon et al., (2016). As was stated, a diffusion of NPs into spheroids is a key factor determining improved nanodrugs-mediated cytotoxicity. A possible explanation was attributed to the loss of proliferative layer during treatment conducting to the reactivation of quiecent cells in spheroids (Solomon et al., 2016).

Increase of photocytotoxicity is correlated with an increment of percentage of apoptosis. After irradiation, Foslip^®^ and mTHPC-EVs induced 1.5–2 times more apoptosis in spheroids compared to free mTHPC (Supplementary Material Table S1). It was already demonstrated that nanovectorized formulations lead to a loss of membrane integrity of spheroid cells (Solomon et al., 2016) involving membrane damage and triggering apoptotic cell death (Gaio et al., 2016).

## Conclusions

EVs have been newly introduced as a novel strategy for the delivery of PSs at tumor sites. As demonstrated in this study mTHPC delivery with EVs to 3D tumor models, evaluated in terms of accumulation and penetration leaves far beyond liposomal mTHPC. The putative mechanism could be related to the unique behavior of mTHPC-EVs in plasma. Taking as a whole, loading of EVs with mTHPC may provide enhanced drug delivery, thus decreasing toxicity and diminishing side effects, and as such representing the future for PDT of cancer.
